# Role of Nutrition in the Etiopathogenesis and Prevention of Nonalcoholic Fatty Liver Disease (NAFLD) in a Group of Obese Adults

**DOI:** 10.3390/medicina59030638

**Published:** 2023-03-22

**Authors:** Daniela Metro, Martina Buda, Luigi Manasseri, Francesco Corallo, Davide Cardile, Viviana Lo Buono, Angelo Quartarone, Lilla Bonanno

**Affiliations:** 1 Department of Biomedical and Dental Sciences and Morphofunctional Imaging, University of Messina, 98122 Messina, Italy; 2 Department Oncological D.A.I., UOC of General Surgery—Oncology, 98125 Messina, Italy; 3 IRCCS Centro Neurolesi Bonino-Pulejo, S.S. 113 Via Palermo, C. da Casazza, 98124 Messina, Italy

**Keywords:** NAFLD, micronutrients, Mediterranean diet, transaminases, prevention, cognitive impairment, cognitive rehabilitation

## Abstract

Nonalcoholic fatty liver disease (NAFLD) is liver damage characterized by an accumulation of triglycerides in hepatocytes of >5% (due to an alteration of the balance of the lipid metabolism in favour of lipogenesis compared to lipolysis) that is not induced by the consumption of alcohol. The pathology includes simple steatosis and nonalcoholic steatohepatitis, or NASH (steatosis associated with microinflammatory activities), which can evolve in 15% of subjects with hepatic fibrosis to cirrhosis and the development of hepatocellular carcinoma. The aim of this study is to report the role of macro- and micronutrients in the pathogenesis and prevention of NAFLD in obese subjects. A total of 22 obese or overweight patients with hepatic steatosis were monitored periodically, evaluating their eating habits, fasting glycaemia, lipid picture, liver enzymes, anthropometric parameters, nutrition status, liver ultrasound, oxidative stress, and adherence to the Mediterranean diet. A statistical analysis shows a significant positive relationship between total cholesterol and the Mediterranean adequacy index (MAI) (r = −0.57; *p* = 0.005) and a significant negative relationship between ALT transaminases and the MAI (r = −0.56; *p* = 0.007). Nutrition and diet are important factors in the pathogenesis and prevention of NAFLD. The dietary model, based on the canons of the Mediterranean diet, prevents and reduces the accumulation of fat in hepatocytes. Therefore, in agreement with other studies in the literature, we can state that a dietary model characterized by foods rich in fibre, carotenoids, polyphenols, ω3 fatty acids, folic acid, and numerous other molecules is inversely correlated with the serum levels of ALT transaminases, an enzyme whose level increases when the liver is damaged and before the most obvious symptoms of organ damage appear.

## 1. Introduction

Nonalcoholic fatty liver disease is liver damage characterized by an accumulation of triglycerides in hepatocytes of >5% (due to an alteration of the balance of the lipid metabolism in favour of lipogenesis compared to lipolysis) that is not induced by the consumption of alcohol or by other etiological factors that cause liver disease, including drugs, toxins, infectious diseases, etc. [1]. NAFLD includes simple steatosis and nonalcoholic steatohepatitis, or NASH (steatosis associated with microinflammatory activities), which can evolve in 15% of subjects with hepatic fibrosis to cirrhosis and the development of hepatocellular carcinoma [2]. In fact, NAFLD progresses in four stages, which includes, in the first stage, the deposition of fat in the liver (NAFL, or nonalcoholic fatty liver). The second stage is characterized by excess fat storage in the liver and NASH (nonalcoholic steatohepatitis) inflammation. Persistent inflammation can cause a scar to form in the liver: this stage is called fibrosis (third stage). The fourth stage is cirrhosis, which is the most severe form of NAFLD, with impaired liver cell structure and function [3]. NAFLD is associated with age, gender, race, and ethnicity. Several genetic and epigenetic factors, a sedentary lifestyle, sleep (sleep apnoea syndrome), and diet composition, may play a role in the pathogenesis of NAFLD and NASH [4,5,6].

NAFLD is frequently associated with obesity, type II diabetes [7,8], dyslipidaemia (hypertriglyceridemia, rather than hypercholesterolemia, and low HDL levels), hyperinsulinemia, and insulin resistance and, more generally, with metabolic syndrome [9]. In most cases, NAFLD is considered to be the hepatic expression of “metabolic syndrome”, a set of clinical conditions that have insulin resistance as a common etiopathogenetic denominator. Metabolic syndrome is characterized by the presence, in the same patient, of abdominal obesity with a circumference of >102 cm in men and of >88 cm in women in addition to two or more of the following parameters: fasting hyperglycaemia, hypertriglyceridemia, reduced HDL cholesterol, and arterial hypertension. The diagnostic process includes the calculation of the body mass index (BMI) and the measurement of the waist. In fact, most patients with NAFLD/NASH have visceral obesity, which favours the establishment of insulin resistance, an important pathogenetic factor of NAFLD and NASH [10]. Its role in the pathogenesis of NAFLD is mainly linked to the ability to promote the accumulation of fat in hepatocytes. Finally, in NAFLD there is an increase in transaminases. ALTs are higher than ASTs, with an ALT/AST ratio > 1, which is contrary to what occurs in alcoholic liver diseases. Since nutrition is an important pathogenetic factor in NAFLD/NASH, the Mediterranean diet seems to be the nutrition model to follow. Several studies indicate that, due to its food’s richness in fibre, carotenoids, omega-3, and folic acid, it is inversely correlated with serum transaminase levels, insulin resistance, and the severity of chronic liver disease and that it would appear to prevent NAFLD [10,11,12,13,14,15,16,17,18].

NAFLD is a multisystem disease, and several clinical features of this disease have been linked to cognitive disorders [19] to the extent that they are considered to be complications of this condition [20,21]. It is not yet clear whether it is NAFLD that causes these cognitive disorders, but we do know that associated conditions, such as vascular dysfunction, systemic inflammation, obstructive sleep apnoea, and atherosclerosis, contribute to cognitive dysfunction [22,23]. Cognitive deficits would appear to affect psychomotor speed, visuospatial functions [24], attention, memory [25], executive functions, and abstract reasoning [26]. It would seem that an influence of the disorder on the cognitive sphere is more likely in women than in men but only if a highly inflammatory state is present [27]. In addition to cognitive effects, NAFLD appears to affect mood by causing depression and anxiety [20,28], but these results as well as those for the cognitive sphere [29] are rather equivocal.

Food intake also has an effect on cognitive performance in patients with a high risk of NAFLD: higher nutrient intake and better diet quality correlate with a better immediate and delayed memory [30]. The role of macro- and micronutrients in the pathogenesis and prevention of NAFLD in obese subjects is reported in the present study. Moreover, active meal management activities are proposed, and their effects on cognitive functions have been measured.

## 2. Materials and Methods

All patients involved within this study were administered Montreal Cognitive Assessment (MoCA) to examine their cognitive profile. MoCA is a rapid tool for the assessment of cognitive functioning. It assesses several cognitive domains: attention and concentration, executive functions, memory, language, visuoconstructive skills, abstraction, calculation, and orientation. The administration time of this test is 10 min, and scores range from 0 to 30, with a score of 26 and above generally considered normal. Since cognitive impairment could affect adherence to treatment and the treatment itself, patients who scored below 26 were not taken into account in the study. After the test’s administration, 22 obese or overweight patients with a mean age of 43 years (range 27–64 years), who were teetotal, who were nonsmoking, and who had hepatic steatosis evidenced by hepatic ultrasonography, were found to be eligible for recruitment. These patients were monitored periodically for six months.

The following were evaluated:

- Eating habits;
- Blood chemistry tests (fasting glycaemia, lipid picture (total cholesterol and HDL cholesterol), and liver enzymes (AST and ALT transaminases));
- Anthropometric parameters (body weight, height, BMI calculation, and waist circumference);
- Nutrition status (bioimpedance test, FM (fat mass), and FFM (lean mass));
- Liver ultrasound;
- Oxidative stress;
- Adherence to the Mediterranean diet.

Patients were also subjected to a low-calorie diet (1300–1400 Kcal), the total caloric intake (TCI) consisting of 20% protein, 25% lipids, and 55% carbohydrates. The dietary recommendations for patients with NAFLD are as follows:

(1) Carbohydrates should be 45–60% of TCI (simple carbohydrates < 10% of TCI);
(2) Lipids should be 20–35% of TCI (2–4 g/day of omega-3);
(3) Proteins should be 20–25% of TCI.

The indicators of oxidative stress, analysed in plasma before and after the administration of the diet, were malonylaldehyde (MDA) and reduced glutathione (GSH). In particular, the oxidative damage, caused by radical species, was evaluated by quantifying the lipid peroxidation expression of oxidative damage by determining the plasma levels of MDA. The modulation of antioxidant defences was evaluated by analysing the plasma levels of reduced glutathione.

The analytical procedures used for the determination of the above parameters have been described in our previous work [31,32]. Adherence to the Mediterranean diet was calculated using the MAI (Mediterranean adequacy index) according to the method of Fidanza et al. [33]. The MAI was calculated from 18 food groups, was expressed as a percentage of the total energy consumed, and was obtained by dividing the sum of the percentages of total energy of food groups typical of the Mediterranean diet (bread, cereals, legumes, potatoes, vegetables, fresh fruits, nuts, fish, wine, and olive oil) by the percentage of total energy of food groups less typical of the Mediterranean diet (milk, dairy products, meat, eggs, animal fats and margarines, sweet drinks, sweets, and sugar).

Specifically, the formula used was

$$MAI=\frac{\%\;of\;energy\;bread+cereals+legumes\;dry\;and\;fresh+potatoes+vegetables+fresh\;fruit+nuts+fish+wine+vegetables\;oils}{\%\;of\;energy\;milk+dairy\;products+meat+eggs+animal\;fats\;and\;margarine+sweet\;beverages+cakes+pies\;and\;cookies+sugar}$$

The value of the MAI for the Italian Mediterranean population was between 4.0 and 8.5.

Adherence to the Mediterranean diet was ensured through regular monitoring of the food diary filled out by patients. Indeed, the detection of eating habits and individual food consumption was carried out by administering questionnaires and using a reasoned nutrition atlas and by executing the following:

(a) Recording reminder of the foods eaten during meals in a day by means of a quantitative assessment;
(b) Registering remembrance of foods consumed habitually and recently through quantitative assessment and registering frequency of consumption.

The consumption of the various food groups consumed was quantified in terms of grams per day. It was therefore possible to trace the daily calorie intake.

We also decided to assess whether the use of this reasoned atlas combined with the person’s active involvement in managing their own meals influenced cognitive performance. Activities such as compilation of diaries, preparing meals or snacks, researching and using recipes, deciding on and organising the daily menu, and preparing shopping lists are often included in ecological rehabilitation programmes. Although these aspects are often taken for granted, these complex skills require the use and coordination of several cognitive abilities [34,35,36]. They enhance personal and instrumental autonomy; may increase flexible thinking; and stimulate memory, attention, and visuospatial abilities [37,38]. Evidence suggests that changing these health behaviours can benefit cognitive function [39,40]. Therefore, at the end of the diet, MoCA was readministered to assess any changes.

## 3. Statistical Analysis

The continuous variables were expressed as the mean ± the standard deviation, whereas the categorical variables were expressed as frequencies and percentages. A paired t-test or Wilcoxon signed-rank test was used to determine if there was a difference between T0 (baseline) and T1 (after 6 months). The correlations between the variables were computed using Spearman’s coefficient or Pearson correlation. Analyses were performed using an open-source R3.0 software package. A 95% confidence level was set with a 5% alpha error. Statistical significance was set at *p* < 0.05.

## 4. Results

**Figure 1:**
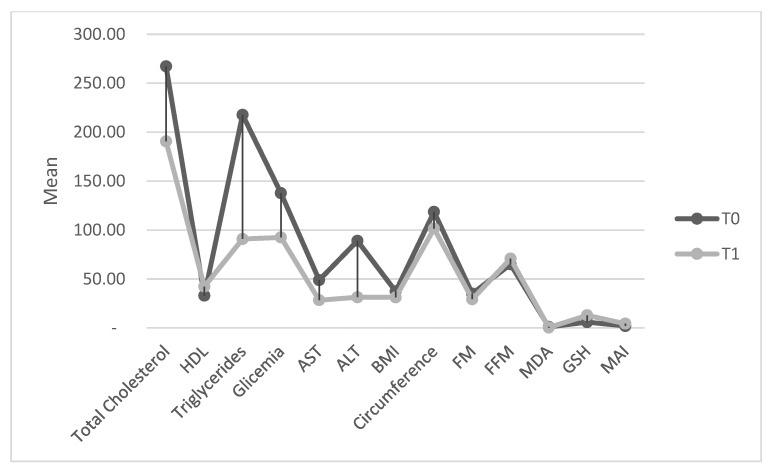
Scatter plots of clinical variables with lines linking the paired T0 and T1.

The demographic and clinical characteristics of the sample are reported in Table 1. The comparison between T0 and T1 showed highlighted significant differences in the clinical variables (*p* < 0.01) (Table 1; Figure 1).

**Figure 2:**
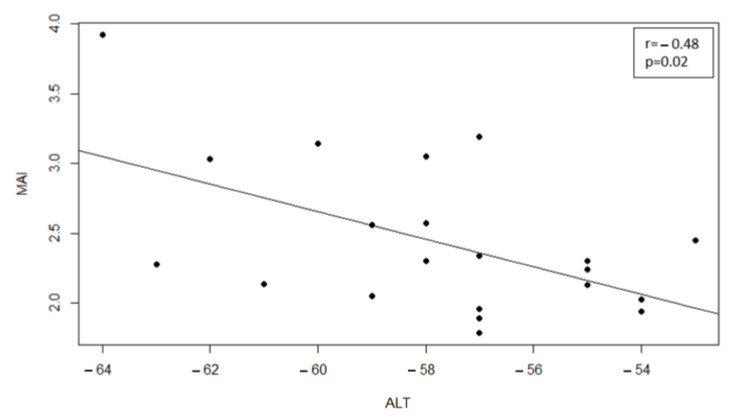
Correlation between clinical scores. Scatter plot of ALT and MAI.

The patients did not show a significant improvement in cognitive performance, measured with the MoCA, between T0 and T1. Spearman correlation showed a significant negative relationship between ALT and MAI (r = −0.48; *p* = 0.02) (Figure 2).

## 5. Discussion

Nutrition and diet are important factors in the pathogenesis and prevention of NAFLD. Numerous studies indicate that the eating habits of NAFLD patients differ significantly from control populations. Among micronutrients, diets rich in fats and carbohydrates play a role in the pathogenesis, prevention, and treatment of NAFLD [41,42,43,44]. Simple sugars, sucrose, and fructose, among carbohydrates [45,46], as well as saturated fatty acids (SFAs) [47,48], trans fatty acids [49], and ω6 fatty acids [50] play a role in the pathogenesis of NAFLD. Dietary models typical of the “Western Diet”, consisting of a combination of highly processed foods, sweets, and drinks enriched with simple sugars as well as red meats and refined cereals, are also considered risk factors for the onset and progression of the disease [51,52]. On the other hand, dietary fibre, foods containing carbohydrates with a low glycaemic index [53,54], as well as monounsaturated fatty acids (MUFAs) and ω3 fatty acids have beneficial effects against NAFLD.

Among the micronutrients, vit. C [27], vit. E [55,56,57], vit. D [31], polyphenols [58,59], and antioxidant molecules prevent the pathogenesis of NAFLD.

Recent scientific testimonies highlight that the increasing use of fructose has favoured the onset and spread of metabolic syndrome and, in particular, of diabetes and that it plays a particularly important role in the development of NAFLD by favouring fibrosis and the progression of NASH [60]. The fructose contained in fruit and honey is present together with glucose and sucrose. By consuming fruit, you also take in fibre, vitamins, and antioxidants, which attenuate the effects of simple sugars on the liver. Fructose can also be used by the food industry as a sweetener in some products, such as drinks and snacks, especially those intended for children. Scientific data have highlighted the risk, linked to its high lipogenetic power, of promoting the onset of insulin resistance. In fact, the increase in the use of beverages rich in fructose increases fat mass, lipogenesis, and inflammation and induces insulin resistance and hypertriglyceridemia, particularly in overweight people [61,62]. It was also observed that, in rats, a diet rich in sucrose resulted in the stimulation of lipogenesis and related enzymatic activities in the liver [63]. Additionally, ω3 fatty acids are important regulators of the genes responsible for the degenerative metabolic pathway of fatty acids and determine the downregulation of the synthesis and deposit of triglycerides [64]. Some studies have shown that ω3 fatty acids reduce hepatic steatosis, improve insulin resistance, and reduce inflammation markers [65].

The dietary model, based on the canons of the Mediterranean diet, prevents and reduces the accumulation of fat in hepatocytes. Observational and experimental studies have affirmed that this dietary model, characterized by foods rich in fibre, carotenoids, polyphenols, ω3 fatty acids, folic acid, and numerous other molecules, is inversely correlated with the serum levels of transaminases, insulin resistance, and the severity of chronic liver diseases. The Mediterranean diet (MD) is not only associated with a low incidence of some chronic degenerative diseases, cancer mortality, and cardiovascular disease; in fact, observational studies and clinical experiences have also highlighted a preventive role against obesity and type II diabetes. The traditional MD is characterized by plant foods: in fact, it is rich in wheat, cereals (including bread and pasta), legumes, fresh fruits, vegetables, nuts, and olive oil. Red meats (beef and lamb) and meat derivatives must be consumed in low quantities. The consumption of fish and chicken is moderate; alcohol (red wine) is consumed in moderate quantities during meals. There is moderate consumption of milk and dairy products as well as animal fats in the form of butter; lard and cream are not included in the diet. The nutrients in the MD that show potentially protective effects are (a) complex carbohydrates, (b) fibres, (c) monounsaturated fatty acids (ω3), (d) polyunsaturated fatty acids (ω3), and (e) bioactive compounds. Bioactive compounds are substances, which are almost all of vegetable origin, that are capable of modulating biological activities and important physiological functions. In fact, multiple effects are attributed to these compounds: antioxidants, anti-inflammatories, the modulation of detoxification enzymes, and the stimulation of the immune system. The main bioactive compounds present in food are (a) carotenoids, (b) polyphenols, and (c) vitamins (vitamins C and E). Furthermore, several studies have shown that MD foods as a whole are more important for the longevity of individual nutrients.

The MD, however, is not characterized by a simple table of foods contained in the classic pyramid developed in the nineties, but it is, above all, a lifestyle that has as its keywords seasonality, conviviality, tradition, frugality, zero waste, and physical activity. This can ensure one’s well-being, longevity, and sustainability. After all, the meaning of the word diet, from the Ancient Greek word dìaita, meaning “lifestyle”, is the search for a balance with oneself, with others, and the environment: a search for well-being that reconciles taste and health.

Recent studies have shown that adherence to the MD tends to decrease, especially in overweight and obese subjects [66]. Several factors contribute to the development and progression of NASH and fibrosis: insulin resistance, hyperinsulinemia, mitochondrial dysfunction, oxidative stress, lipid peroxidation, an increased production of proinflammatory cytokines, hepatic stellate cell activation, and apoptosis [67]. Hepatic stellate cells induce fibrosis and collagen deposition [68].

Oxidative stress (alterations in the balance between prooxidant and antioxidant activities) is due to an increase in reactive oxygen species (ROS). The oxidation of fatty acids is an active source of ROS. This increase in ROS produces damage to DNA and proteins, damages the structure and function of membranes through lipid peroxidation, and increases the release of proinflammatory cytokines [69,70,71,72]. Finally, the results of various experimental and clinical studies suggest that oxidative stress is associated with many of the components of metabolic syndrome, diabetes mellitus, hypertension, obesity, dyslipidaemia, and inflammation [73], and an inverse association between the Mediterranean diet and prevalence is also demonstrated for metabolic syndrome [74].

## 6. Conclusions

The results obtained from our investigation would seem to suggest that an adequate diet, conforming to the characteristics of the Mediterranean diet, could have a modulatory effect on the prevention, development, and progression of NAFLD. Further studies could investigate more deeply the extent and quality of these effects on a larger sample.

## Figures and Tables

**Table 1:** Demographic and clinical characteristics of sample.

	T0	T1	
Variables	Mean ± SD	Mean ± SD	*p*-Student
**Age**	43.0 ± 12.40	-	-
**Education**	14.19 ± 3.42	-	-
**MoCA**	27.2 ± 1.23	27.41 ± 1.37	0.13
Total Cholesterol	267.14 ± 12.87	190.55 ± 121.43	<0.001 *^±^
HDL	33.00 ± 3.18	42.27 ± 4.42	<0.001 *
Triglycerides	217.64 ± 9.04	90.86 ± 4.99	<0.001 *^±^
Glycaemia	137.68 ± 8.92	92.45 ± 4.30	<0.001 *
AST	48.82 ± 2.82	28.32 ± 3.05	<0.001 *
ALT	88.91 ± 3.89	31.32 ± 2.82	<0.001 *
BMI	37.36 ± 2.57	31.23 ± 2.35	<0.001 *
Circumference	118.55 ± 2.56	101.45 ± 2.97	<0.001 *^±^
FM	35.05 ± 3.46	29.27 ± 2.71	<0.001 *
FFM	64.95 ± 3.46	70.73 ± 2.71	<0.001 *
MDA	0.98 ± 0.15	0.36 ± 0.07	<0.001 *
GSH	5.90 ± 0.55	12.89 ± 1.37	<0.001 *
MAI	1.92 ± 0.14	4.34 ± 0.46	<0.001 *^±^

^±^ Wilcoxon-signed rank test. * Statistically significant.

## Data Availability

Data can be found in this database: https://doi.org/10.5281/zenodo.7741268.

## References

[1] Oldfield D.R., Johnson D. (2015). Non-alcoholic fatty liver disease and the gut micribiota:exploringthe connection. Gastro. Open J..

[2] Rinella M.E. (2015). Nonalcoholic fatty liver disease: A systematic review. JAMA.

[3] Schattenberg J.M., Bergheim I. (2019). Nutritional Intake and the Risk for Non-Alcoholic Fatty Liver Disease (NAFLD). Nutrients.

[4] Nobili V., Svegliati-Baroni G., Alisi A., Miele L., Valenti L., Vajro P. (2013). A 360-degree overview of paediatric NAFLD: Recent insights. J. Hepatol..

[5] Africa J.A., Newton K.P., Schwimmer J.B. (2016). Lifestyle Interventions Including Nutrition, Exercise, and Supplements for Nonalcoholic Fatty Liver Disease in Children. Dig. Dis. Sci..

[6] Katsagoni C.N., Georgoulis M., Papatheodoridis G.V., Fragopoulou E., Ioannidou P., Papageorgiou M., Alexopoulou A., Papadopoulos N., Deutsch M., Kontogianni M.D. (2017). Associations Between Lifestyle Characteristics and the Presence of Nonalcoholic Fatty Liver Disease: A Case-Control Study. Metab. Syndr. Relat. Disord..

[7] Angulo P. (2002). Nonalcoholic fatty liver disease. N. Engl. J. Med..

[8] Moscatiello S., Manini R., Marchesini G. (2007). Diabetes and liver disease: An ominous association. Nutr. Metab. Cardiovasc. Dis..

[9] Papatheodoridi M., Cholongitas E. (2018). Diagnosis of Non-alcoholic Fatty Liver Disease (NAFLD): Current Concepts. Curr. Pharm. Des..

[10] Harrison S.A., Torgerson S., Hayashi P.H. (2003). The natural history of nonalcoholic fatty liver disease: A clinical histopathological study. Am. J. Gastroenterol..

[11] Marchesini G., Marzocchi R., Agostini F., Bugianesi E. (2005). Nonalcoholic fatty liver disease and the metabolic syndrome. Curr. Opin. Lipidol..

[12] Clark J.M. (2006). The epidemiology of nonalcoholic fatty liver disease in adults. J. Clin. Gastroenterol..

[13] Lazo M., Clark J.M. (2008). The Epidemiology of Nonalcoholic fatty liver Disease: A global Perspective. Semin. Liver Dis..

[14] Morisco F., Vitaglione P., Amoruso D., Russo B., Fogliano V., Caporaso N. (2008). Food and liver health. Mol. Asp. Med..

[15] Fan J.G., Jia J.D., Li Y.M., Wang B.Y., Lu L.G., Shi J.P., Chan L.Y. (2011). Chinese Association for the Study of Liver Disease. Guidelines for the diagnosis and management of nonalcoholic fatty liver didease: Update 2010. J. Dig. Dis..

[16] Caporaso N., Morisco F., Camera S., Graziani G., Donnarumma L., Ritieni A. (2012). Dietary approach in the prevention and treatment of NAFLD. Front. Biosci..

[17] European Association for the Study of the Liver (EASL) (2016). EASL-EASD-EASO Clinical Practice Guidelines for the management of non-alcoholic fatty liver disease. J. Hepatol..

[18] Rahim U., Naveed R., Ghulam N., Hamid U., Yi S., Yu-Dong Z., Junfen F. (2019). Role of nutrition in the Phatogenesis and Prevention of Non-alcoholic Fatty Liver Disease: Recent Updates. Int. J. Biol. Sci..

[19] Kjærgaard K., Mikkelsen A.C.D., Wernberg C.W., Grønkjær L.L., Eriksen P.L., Damholdt M.F., Mookerjee R.P., Vilstrup H., Lauridsen M.M., Thomsen K.L. (2021). Cognitive Dysfunction in Non-Alcoholic Fatty Liver Disease-Current Knowledge, Mechanisms and Perspectives. J. Clin. Med..

[20] Colognesi M., Gabbia D., De Martin S. (2020). Depression and Cognitive Impairment-Extrahepatic Manifestations of NAFLD and NASH. Biomedicines.

[21] Lombardi R., Fargion S., Fracanzani A.L. (2019). Brain involvement in non-alcoholic fatty liver disease (NAFLD): A systematic review. Dig. Liver Dis..

[22] Khanna S., Parikh N.S., VanWagner L.B. (2022). Fatty liver and cerebrovascular disease: Plausible association and possible mechanisms. Curr. Opin. Lipidol..

[23] Frisardi V., Solfrizzi V., Seripa D., Capurso C., Santamato A., Sancarlo D., Vendemiale G., Pilotto A., Panza F. (2010). Metaboliccognitive syndrome: A cross-talk between metabolic syndrome and Alzheimer’s disease. Ageing Res. Rev..

[24] Weinstein A.A., de Avila L., Paik J., Golabi P., Escheik C., Gerber L., Younossi Z.M. (2018). Cognitive Performance in Individuals with Non-Alcoholic Fatty Liver Disease and/or Type 2 Diabetes Mellitus. Psychosomatics.

[25] Seo S.W., Gottesman R.F., Clark J.M., Hernaez R., Chang Y., Kim C., Ha K.H., Guallar E., Lazo M. (2016). Nonalcoholic fatty liver disease is associated with cognitive function in adults. Neurology.

[26] Weinstein G., Davis-Plourde K., Himali J.J., Zelber-Sagi S., Beiser A.S., Seshadri S. (2019). Non-alcoholic fatty liver disease, liver fibrosis score and cognitive function in middle-aged adults: The Framingham Study. Liver Int. Off. J. Int. Assoc. Study Liver.

[27] Kang S., Kim E., Cho H., Kim D.J., Kim H.C., Jung S.J. (2022). Associations between non-alcoholic fatty liver disease and cognitive impairment and the effect modification of inflammation. Sci. Rep..

[28] Filipović B., Marković O., Đurić V., Filipović B. (2018). Cognitive Changes and Brain Volume Reduction in Patients with Nonalcoholic Fatty Liver Disease. Can. J. Gastroenterol. Hepatol..

[29] Gerber Y., VanWagner L.B., Yaffe K., Terry J.G., Rana J.S., Reis J.P., Sidney S. (2021). Non-alcoholic fatty liver disease and cognitive function in middle-aged adults: The CARDIA study. BMC Gastroenterol..

[30] Tan S.Y., Georgousopoulou E.N., Cardoso B.R., Daly R.M., George E.S. (2021). Associations between nut intake, cognitive function and non-alcoholic fatty liver disease (NAFLD) in older adults in the United States: NHANES 2011-14. BMC Geriatr..

[31] Metro D., Cernaro V., Santoro D., Papa M., Buemi M., Benvenga S., Manasseri L. (2017). Beneficial effects of oral pure caffeine on oxidative stress. J. Clin. Transl. Endocrinol..

[32] Metro D., Tardugno R., Papa M., Bisignano C., Manasseri L., Calabrese G., Gervasi T., Dugo G., Cicero N. (2018). Adherence to the Mediterranean diet in a Sicilian student population. Nat. Prod. Res..

[33] Alberti-Fidanza A., Fidanza F. (2004). Mediterranean Adequacy Index of Italian diets. Public Health Nutr..

[34] Schultheis H., Cooper R.P. (2022). Everyday Activities. Top Cogn. Sci..

[35] Mis R., Giovannetti T. (2022). Similarities between Cognitive Models of Language Production and Everyday Functioning: Implications for Development of Interventions for Functional Difficulties. Top Cogn. Sci..

[36] Pinard S., Bottari C., Laliberté C., Pigot H., Olivares M., Couture M., Giroux S., Bier N. (2021). Design and usability evaluation of COOK, an assistive technology for meal preparation for persons with severe TBI. Disabil. Rehabil. Assist. Technol..

[37] Gibson E., Koh C.L., Eames S., Bennett S., Scott A.M., Hoffmann T.C. (2022). Occupational therapy for cognitive impairment in stroke patients. Cochrane Database Syst. Rev..

[38] Abraha I., Rimland J.M., Trotta F.M., Dell’Aquila G., Cruz-Jentoft A., Petrovic M., Gudmundsson A., Soiza R., O’Mahony D., Guaita A. (2017). Systematic review of systematic reviews of non-pharmacological interventions to treat behavioural disturbances in older patients with dementia. The Senator-OnTop series. BMJ Open.

[39] Martin A., Booth J.N., Laird Y., Sproule J., Reilly J.J., Saunders D.H. (2018). Physical activity, diet and other behavioural interventions for improving cognition and school achievement in children and adolescents with obesity or overweight. Cochrane Database Syst. Rev..

[40] Valls-Pedret C., Sala-Vila A., Serra-Mir M., Corella D., de la Torre R., Martínez-González M.Á., Martínez-Lapiscina E.H., Fitó M., Pérez-Heras A., Salas-Salvadó J. (2015). Mediterranean Diet and Age-Related Cognitive Decline: A Randomized Clinical Trial. JAMA Intern. Med..

[41] Metro D., Papa M., Manasseri L., Gervasi T., Campone L., Pellizzeri V., Tardugno R., Dugo G. (2020). Mediterranean diet in a Sicilian student population. Second part: Breakfast and its nutritional profile. Nat. Prod. Res..

[42] Semiane N., Foufelle F., Ferré P., Hainault I., Ameddah S., Mallek A., Khalkhal A., Dahmani Y. (2017). High carbohydrate diet induces nonalcoholic steato-hepatitis (NASH) in a desert gerbil. Comptes Rendus Biol..

[43] Neuschwander T., Brent A. (2013). Carbohydrate intake and nonalcoholic fatty liver disease. Curr. Opin. Clin. Nutr. Metab. Care.

[44] Shi H., Fu J., Wang C. (2008). Clinical value of hepatic fibrosis parameters and serum ferritin in obese children with nonalcoholic fatty liver disease. J. Zhejiang Univ. Med. Sci..

[45] Kargulewicz A., Stankowiak-Kulpa H., Grzymisławski M. (2014). Dietary recommendations for patients with nonalcoholic fatty liver disease. Prz. Gastroenterol..

[46] Roglans N., Vilà L., Farré M., Alegret M., Sánchez R.M., Vázquez-Carrera M., Laguna J.C. (2007). Impairment of hepatic Stat-3 activation and reduction of PPARalpha activity in fructose-fed rats. Hepatology.

[47] Lê K.A., Bortolotti M. (2008). Role of dietary carbohydrates and macronutrients in the pathogenesis of nonalcoholic fatty liver disease. Curr. Opin. Clin. Nutr. Metab. Care.

[48] Wang D., Wei Y.R., Pagliassotti M.J. (2006). Saturated fatty acids promote endoplasmic reticulum stress and liver injury in rats with hepatic steatosis. Endocrinology.

[49] Lopez-Garcia E., Schulze M.B., Meigs J.B., Manson J.E., Rifai N., Stampfer M.J., Willett W.C., Hu F.B. (2005). Consumption of trans fatty acids is related to plasma biomarkers of inflammation and endothelial dysfunction. J. Nutr..

[50] Reiner Ž., Catapano A.L., De Backer G., Graham I., Taskinen M.R., Wiklund O., Agewall S., Alegría E., Chapman M.J., Durrington P. (2011). Clinical Practice Guidelines Committee of the Spanish Society of Cardiology. Rev. Esp. Cardiol..

[51] Cordain L., Eaton S.B., Sebastian A., Mann N., Lindeberg S., Watkins B.A., O’Keefe J.H., Brand-Miller J. (2005). Origins and evolution of the Western diet: Health implications for the 21st century. Am. J. Clin. Nutr..

[52] Zelber-Sagi S., Ivancovsky-Wajcman D., Fliss Isakov N., Webb M., Orenstein D., Shibolet O., Kariv R.J. (2018). High red and processed meat consumption is associated with non-alcoholic fatty liver disease and insulin resistance. Hepatology.

[53] Anderson J.W., Randles K.M., Kendall C.W., Jenkins D.J. (2004). Carbohydrate and fiber recommendations for individuals with diabetes: A quantitative assessment and meta-analysis of the evidence. J. Am. Coll. Nutr..

[54] Brighenti F., Benini L., Del Rio D., Casiraghi C., Pellegrini N., Scazzina F., Jenkins D.J., Vantini I. (2006). Colonic fermentation of indigestible carbohydrates contributes to the second-meal effect. Am. J. Clin. Nutr..

[55] Harrison S.A., Torgerson S., Hayashi P., Ward J., Schenker S. (2003). Vitamin E and vitamin C treatment improves fibrosis in patients with nonalcoholic steatohepatitis. Am. J. Gastroenterol..

[56] Lavine J.E., Schwimmer J.B., Van Natta M.L., Molleston J.P., Murray K.F., Rosenthal P., Abrams S.H., Scheimann A.O., Sanyal A.J., Chalasani N. (2011). Effect of vitamin E or metformin for treatment of nonalcoholic fatty liver disease in children and adolescents: The TONIC randomized controlled trial. Nonalcoholic Steatohepatitis Clinical Research Network. JAMA.

[57] Nobili V., Manco M., Devito R., Ciampalini P., Piemonte F., Marcellini M. (2006). Effect of vitamin E on aminotransferase levels and insulin resistance in children with non-alcoholic fatty liver disease. Aliment. Pharmacol. Ther..

[58] Vajro P., Mandato C., Franzese A., Ciccimarra E., Lucariello S., Savoia M., Capuano G., Migliaro F.J. (2004). Vitamin E treatment in pediatric obesity-related liver disease: A randomized study. Pediatr. Gastroenterol. Nutr..

[59] Sharifi N., Amani R., Hajiani E., Cheraghian B. (2014). Does vitamin D improve liver enzymes, oxidative stress, and inflammatory biomarkers in adults with non-alcoholic fatty liver disease? A randomized clinical trial. Endocrine.

[60] Faghihzadeh F., Adibi P., Rafiei R., Hekmatdoost A. (2014). Resveratrol supplementation improves inflammatory biomarkers in patients with nonalcoholic fatty liver disease. Nutr. Res..

[61] Chen S., Zhao X., Ran L., Wan J., Wang X., Qin Y., Shu F., Gao Y., Yuan L., Zhang Q. (2015). Resveratrol improves insulin resistance, glucose and lipid metabolism in patients with non-alcoholic fatty liver disease: A randomized controlled trial. Dig. Liver Dis..

[62] Lim J.S., Mietus-Snyder M., Valente A., Schwarz J.M., Lustig R.H. (2010). The role of fructose in the pathogenesis of NAFLD and the metabolic syndrome. Nat. Rev. Gastroenterol. Hepatol..

[63] Aldámiz-Echevarría L., de Las Heras J., Couce M.L., Alcalde C., Vitoria I., Bueno M., Blasco-Alonso J., Concepción García M., Ruiz M., Suárez R. (2020). Non-alcoholic fatty liver in hereditary fructose intolerance. Clin. Nutr..

[64] Cantoral A., Contreras-Manzano A., Luna-Villa L., Batis C., Roldán-Valadez E.A., Ettinger A.S., Mercado A., Peterson K.E., Téllez-Rojo M.M., Rivera J.A. (2019). Dietary Sources of Fructose and Its Association with Fatty Liver in Mexican Young Adults. Nutrients.

[65] Carrozza G., Russo C.R. (1977). Ripercussioni metaboliche nel fegato di ratto dell’alimentazione con saccarosio. Atti Soc. Peloritana Sci. Fis. Matem Nat..

[66] Sinha R.A., Khare P., Rai A., Maurya S.K., Pathak A., Mohan V., Nagar G.K., Mudiam M.K., Godbole M.M., Bandyopadhyay S. (2009). Anti-apoptotic role of omega-3-fatty acids in developing brain: Perinatal hypothyroid rat cerebellum as apoptotic model. Int. J. Dev. Neurosci..

[67] Masterton G.S., Plevris J.N., Hayes P.C. (2010). Review article: Omega-3 fatty acids—A promising novel therapy for non-alcoholic fatty liver disease. Aliment. Pharmacol. Ther..

[68] Aller R., Burgueño Gomez B., Sigüenza R., Fernández-Rodríguez C., Fernández N., Antolín B., Durà M., Pina M., Lorenzo S., García C. (2019). Comparative study of overweight and obese patients with nonalcoholic fatty liver disease. Rev. Esp. Enferm. Dig..

[69] Chitturi S., Farrell G.C. (2001). Etiopathogenesis of nonalcoholic steatohepatitis. Semin. Liver Dis..

[70] Seki S., Kitada T., Yamada T., Sakaguchi H., Nakatani K., Wakasa K. (2002). In situ detection of lipid peroxidation and oxidative DNA damage in non-alcoholic fatty liver diseases. J. Hepatol..

[71] Sreekumar R., Rosado B., Rasmussen D., Charlton M. (2003). Hepatic gene expression in histologically progressive nonalcoholic steatohepatitis. Hepatology.

[72] Greco D., Kotronen A., Westerbacka J., Puig O., Arkkila P., Kiviluoto T., Laitinen S., Kolak M., Fisher R.M., Hamsten A. (2008). Gene expression in human NAFLD. Am. J. Physiol. Gastrointest. Liver Physiol..

[73] Hopps E., Noto D., Caimi G., Averna M.R. (2010). A novel component of the metabolic syndrome: The oxidative stress. Nutr. Metab. Cardiovasc. Dis..

[74] Babio N., Bulló M., Basora J., Martínez-González M.A., Fernández-Ballart J., Márquez-Sandoval F., Molina C., Salas-Salvadó J., Nureta-PREDIMED Investigators (2009). Adherence to the Mediterranean diet and risk of metabolic syndrome and its components. Nutr. Metab. Cardiovasc. Dis..

